# Supplementary material for: Network disruption via continuous batch removal: The case of Sicilian Mafia
Source: PLoS One. 2024 Aug 21;19(8):e0308722. doi: 10.1371/journal.pone.0308722 (PMC11338461; doi:10.1371/journal.pone.0308722)
Supplement: Attachment — Submitted filename: Rebuttal_Letter.pdf [file pone.0308722.s001.pdf]

PONE-D-23-41294
Network disruption via continuous batch removal: The case of Sicilian Mafia
PLOS ONE

# Rebuttal Letter

Dear Editor,

Thank you very much for handling this submission. According to the reviewers' comments and suggestions, we have carefully revised the manuscript.

The main changes include: (i) We have added two more baselines in our experiment, i.e., a community-based approach and a spectral-based approach, and all related result and discussion are revised accordingly; (ii) We have added statistic analysis when comparing the result under the standard setting and that with the constraint of within-LCC. (iii) We have revised the part of future directions to include discussions on other structural characteristics; (iv) We have revised several notations and equations so they become clearer and remain consistent throughout the paper; (v) Our dataset is uploaded to Kaggle, the url is `https://www.kaggle.com/datasets/mingshanjia/sicilian-mafia-criminal-network`. Enclosed please find point-to-point responses to the reviewing reports.

Kind regards,
Mingshan Jia, Pasquale De Meo, Bogdan Gabrys and Katarzyna Musial

---

## Reviewer 1

**Reviewer Comment C 1.1** — I have recommended to work on different dataset instead of using a single dataset.

**Authors response**: We agree that including more datasets would help make the proposed approaches more convincing. However, this article is written as a focused study on the Sicilian Criminal Network, and we provided a deep and thorough study on it, not only experimenting with different disruption strategies, but also providing theoretical analysis. Moreover, our code and experiment settings are open sourced, so it can be easily applied to other networks. We provide two more examples here, one on a criminal network (754 nodes and 2127 edges) [1], another on a power grid network of Chile (218 nodes and 527 edges) [2].

---

## Reviewer 2

**Reviewer Comment C 2.1** — It is noteworthy that all introduced nomenclature should be clearly defined either in the introduction or upon their first mention to facilitate understanding for readers unfamiliar with the terminology.

Table 1: Continuous disruption on a criminal network (batch size $b = 2$, measured in $R$@20%).

| **Strategy** | **Standard** | **W-LCC** |
|---|---|---|
| DEG-BTW | 0.1381 | 0.1239 |
| BTW-DEG | 0.0755 | 0.0744 |
| CI | 0.1470 | 0.1150 |
| CoreHD | 0.1486 | 0.1307 |
| APTA | 0.0921 | 0.0885 |
| MBA | 0.0812 | 0.0744 |
| GND | - | 0.1302 |
| GRD | 0.0633 | 0.0602 |
| SF-GRD | 0.0639 | 0.0672 |

Table 2: Continuous disruption on a power grid network (batch size $b = 2$, measured in $R$@20%).

| **Strategy** | **Standard** | **W-LCC** |
|---|---|---|
| DEG-BTW | 0.2427 | 0.2114 |
| BTW-DEG | 0.1668 | 0.1720 |
| CI | 0.2921 | 0.2235 |
| CoreHD | 0.2804 | 0.2200 |
| APTA | 0.2408 | 0.2404 |
| MBA | 0.1733 | 0.1678 |
| GRD | 0.0633 | 0.0602 |
| GRD | 0.1943 | 0.2156 |
| SF-GRD | 0.1551 | 0.1495 |

**Authors response**: As the reviewer suggested, we have thoroughly checked all the nomenclature, making sure they are clearly defined at their first appearances. Further, we have revised several notations and equations so they become clearer and consistent throughtout the manuscript.

**Reviewer Comment C 2.2** — While they claimed the robustness of their approach, there is a lack of comparative analysis with existing research to substantiate this assertion. To address this gap, a thorough comparison with other relevant works is warranted. This would involve examining how the proposed strategies fare against those from prior studies in terms of effectiveness, efficiency, and robustness under similar conditions.

**Authors response**: As suggested, we have further expanded the baselines by including two more disruption methods in our study, i.e., a community-based approach (MBA) [3] and a spectral-based approach (GND) [4].

- *Module-based Attack (MBA)* [3]. MBA proposes to first identify topological communities within the network using a well established heuristic algorithm for community detection (Louvain algorithm is used in the experiment [5]). The nodes involved in links between different communities are then removed, starting with the one that has the highest betweenness centrality and proceeding in descending order. In order to make it suitable for batch removal, i.e., to dealing with the situation

where the batch size exceeds the number of nodes with inter-community links, we choose to then target nodes based on their degree.

- *Generalised Network Dismantling (GND)* [4]. GND is a spectral-cut based strategy, where the network is dismantled by selectively targeting and removing certain nodes based on their spectral properties. GND operates by leveraging the spectrum of the graph Laplacian, particularly focusing on the Fiedler vector, i.e., the eigenvector associated with the second-smallest eigenvalue of the Laplacian matrix. This vector provides insights into the weak links of a network that, if removed, would lead to the network's fragmentation into disconnected components. GND is shown to be efficient for network dismantling. However, the disruption process is stochastic due to the random nature of spectral operation. Moreover, GND can not be used when network becomes very sparse because there is no meaningful Fiedler vector to work with. We also extend GND for batch removal in the following experiment.

Section 5.1 and Section 5.3 are revised accordingly, together with changes in Table 3 and Table 5 in the manuscript.

**Reviewer Comment C 2.3** — Furthermore, the study proposed a within-LCC constraint simulating police raid operations. This constraint was found to impact the effectiveness of disruption strategies, favoring degree-based approaches with moderate batch sizes over betweenness-based ones. However, to provide a more comprehensive evaluation, additional statistical analysis of the results is necessary. This analysis should include measures such as confidence intervals, significance tests, and potentially sensitivity analyses to assess the robustness of the findings under different conditions.

**Authors response**: Following the reviewer's recommendations, we have incorporated T-Tests to compare the effectiveness of disruption strategies under the standard setting and those applied with the within-LCC constraint. The results of these analyses are detailed in Table 4 of our manuscript. Furthermore, to provide a richer understanding of the statistical significance of our findings, we have also included discussions on the implications of these results, specifically focusing on how the within-LCC constraint influences the performance of degree-based versus betweenness-based disruption strategies.

**Reviewer Comment C 2.4** — Moreover, the authors introduced a structurally-filtered greedy disruption strategy based on the characteristics of nodes removed by a naive greedy approach. This strategy reportedly outperformed classic disruption strategies while maintaining similar time complexity to betweenness centrality. To strengthen the validity of this claim, it is essential to conduct a more rigorous comparison with existing approaches. This could involve assessing the performance of the proposed strategy against a wider range of benchmarks and providing a detailed discussion of the results.

**Authors response**: As recommended by the review, we have broadened our comparison base to include seven different approaches. This expansion allows for a comprehensive evaluation of our Structurally-Filtered Greedy Disruption (SF-GRD) method against a more extensive set of benchmarks. Specifically, Table 6 in our manuscript now presents the outcomes of this enhanced comparison. It showcases the results of the most effective disruption strategy from the expanded set of baselines, and then juxtaposes these findings with the performance of both the Greedy Disruption (GRD) and SF-GRD approaches. This detailed comparison underscores the superiority of the SF-GRD method in disrupting network structures

efficiently, while also maintaining a comparable time complexity to traditional betweenness centrality measures.

**Reviewer Comment C 2.5** — In addition to addressing these points, future research directions could involve exploring how other structural characteristics of networks can offer valuable insights into network disruption. This could lead to a deeper understanding of network dynamics and the development of even more effective disruption strategies.

**Authors response**: we have thoroughly revised the corresponding section of our manuscript to encompass a broader discussion on the exploration of other structural characteristics of networks. Specifically, we delve into the potential of motif-based approaches and the significance of metrics that shed light on the formation of fundamental structures, such as triangles or quadrangles. This expanded discussion aims to highlight how a deeper investigation into these structural elements can offer valuable insights into network disruption tactics, potentially paving the way for the development of more sophisticated and effective disruption strategies.

## References


[1] Kunegis J. Konect: the koblenz network collection. In: Proceedings of the 22nd international conference on world wide web; 2013. p. 1343–1350.

[2] Kim H, Olave-Rojas D, Álvarez-Miranda E, Son SW. In-depth data on the network structure and hourly activity of the central Chilean power grid. Scientific data. 2018;5(1):1–10.

[3] Requião da Cunha B, González-Avella JC, Gonçalves S. Fast fragmentation of networks using module-based attacks. PloS one. 2015;10(11):e0142824.

[4] Ren XL, Gleinig N, Helbing D, Antulov-Fantulin N. Generalized network dismantling. Proceedings of the national academy of sciences. 2019;116(14):6554–6559.

[5] Blondel VD, Guillaume JL, Lambiotte R, Lefebvre E. Fast unfolding of communities in large networks. Journal of statistical mechanics: theory and experiment. 2008;2008(10):P10008.
