# Supplementary material for: Network disruption via continuous batch removal: The case of Sicilian Mafia
Source: PLoS One. 2024 Aug 21;19(8):e0308722. doi: 10.1371/journal.pone.0308722 (PMC11338461; doi:10.1371/journal.pone.0308722)
Supplement: Attachment — Submitted filename: Rebuttal_letter_R2.pdf [file pone.0308722.s002.pdf]

PONE-D-23-41294R1
Network disruption via continuous batch removal: The case of Sicilian Mafia
PLOS ONE

Rebuttal Letter

Dear Editor,

Thank you very much for handling this submission. According to your suggestion and the reviewers' comments, we have carefully revised the manuscript and replied the reviewers' comments.

The main changes include: (i) We have explaind the concept of robustness in more details; (ii) we further explained how the proposed method mitigates this computational difficulty and how it efficiently obtains approximate solutions. Enclosed please find point-to-point responses to the reviewing reports.

Kind regards,
Mingshan Jia, Pasquale De Meo, Bogdan Gabrys and Katarzyna Musial

---

Reviewer 3

Reviewer Comment C 3.1 — Insufficient explanation of robustness: The concept of robustness in network analysis methods is not sufficiently explained in this paper. In particular, it is necessary to clearly demonstrate, with theoretical justification, how the proposed Greedy Disruption Approach (GRD) improves robustness.

Authors response: As suggested by the reviewer, we have added more explanation about the concept of robustness in section 3.1. The experiment result in Table 5 shows that GRD can achieve the optimal R@20% and R@40% when batch size is larger than one.

Reviewer Comment C 3.2 — Dealing with NP-hardness: While it is stated that the network disruption task is NP-hard, the explanation of how this difficulty is addressed is insufficient. In particular, it is necessary to explain in detail how the proposed method mitigates this computational difficulty or how it efficiently obtains approximate solutions.

Authors response: According the reviewer's comment, we further explained how the proposed method mitigates this computational difficulty and how it efficiently obtains approximate solutions. The NP-hardness of the network disruption task is addressed by proposing the SF-GRD approach. The naive greedy approach, while effective, is computationally infeasible for large networks due to its factorial time complexity. To mitigate this, the SF-GRD restricts the search space to nodes that are highly ranked in betweenness and degree centrality and are articulation points, significantly reducing computational complexity. This method achieves a balance between disruption effectiveness and computational efficiency, as shown in the experimental results section (Table 6 and Section 6.2).

Reviewer Comment C 3.3 — Relationship between noise removal and feature reduction: There is a possibility that removing nodes from the network may simultaneously reduce potentially important features. Consideration of this point is lacking. The theoretical basis and experimental verification of how to balance noise removal and retention of important structural information should be shown.

Authors response: The objective of disrupting a criminal network is to break its original robust network structure. An efficient disruption strategy should be able to destroy this structure in fewer steps, minimising the risk of reducing potentially important features. This is why we focus on the initial disruption steps and introduce the partial robustness metric. The partial robustness metric evaluates the impact of removing a small percentage of nodes, ensuring that we effectively target critical nodes while retaining important structural information. This balance between noise removal and feature retention is both theoretically justified and experimentally verified in our study (Section 6.2).

Reviewer Comment C 3.4 — Validity of evaluation metrics: While the reduction in LCC size is used as the main evaluation metric, there is insufficient discussion about whether this metric appropriately reflects the vulnerability of actual criminal networks. It is necessary to explain in more detail the comparison with other possible evaluation metrics and the reason for choosing this metric.

Authors response: LCC size (also R and R@20% which are defined based on it) is chosen for several reasons:

First, the LCC size directly indicates the largest group of interconnected nodes. In the context of criminal networks, maintaining a large LCC means that a significant portion of the network remains functional and coordinated.Disrupting the LCC breaks down this coordination, severely impairing the network's operational capability.

Second, in real-world scenarios, law enforcement aims to fragment criminal networks to hinder communication and coordination among members. A significant reduction in LCC size means that the network is fragmented into smaller, less efficient sub-networks, making it harder for the network to operate effectively.

Third, LCC size is straightforward to measure and interpret compared to other metrics like average path length or modularity. This simplicity allows for clear and direct assessment of the network's vulnerability.

In short, LCC size reduction is chosen because it best captures the overall cohesiveness and operational capability of criminal networks. By focusing on this metric, we ensure that our disruption strategies directly target the network's core structure, leading to effective fragmentation and disruption of criminal activities. It is the standard metric used in most disruption analysis [1–4].

References

[1] Braunstein A, Dall'Asta L, Semerjian G, Zdeborová L. Network dismantling. Proceedings of the National Academy of Sciences. 2016;113(44):12368–12373.

[2] Holme P, Kim BJ, Yoon CN, Han SK. Attack vulnerability of complex networks. Physical review E. 2002;65(5):056109.

[3] Schneider CM, Moreira AA, Andrade Jr JS, Havlin S, Herrmann HJ. Mitigation of malicious attacks on networks. Proceedings of the National Academy of Sciences. 2011;108(10):3838–3841.

[4] Wandelt S, Sun X, Feng D, Zanin M, Havlin S. A comparative analysis of approaches to network-dismantling. Scientific reports. 2018;8(1):13513.
